# Live-Cell Mesothelioma Biobank to Explore Mechanisms of Tumor Progression

**DOI:** 10.3389/fonc.2018.00040

**Published:** 2018-02-23

**Authors:** Kathrin Oehl, Jelena Kresoja-Rakic, Isabelle Opitz, Bart Vrugt, Walter Weder, Rolf Stahel, Peter Wild, Emanuela Felley-Bosco

**Affiliations:** ^1^Department of Pathology and Molecular Pathology, University Hospital Zürich, Zürich, Switzerland; ^2^Laboratory of Molecular Oncology, Division of Thoracic Surgery, University Hospital Zürich, Zürich, Switzerland; ^3^Cancer Center Zürich, University Hospital Zürich, Zürich, Switzerland

**Keywords:** mesothelioma, primary culture, tumor progression, chemoresistance, genetic profiling, mutations, copy number, cisplatin and pemetrexed

## Abstract

Experimental models closely representing *in vivo* conditions allow investigating mechanisms of resistance. Our aims were to establish a live-cell biobank of malignant pleural mesothelioma (MPM) samples and to obtain proof of principle that primary culture chemoresistant models, mimicking tumor progression observed in patients, can be obtained *in vitro*, providing a useful tool to investigate underlying mechanisms. Primary mesothelioma cultures were established from 235 samples between 2007 and 2014. Of two MPM patients, primary cultures obtained at different time points: at initial diagnosis, after neoadjuvant treatment at surgery and/or after tumor recurrence, were deeply investigated. Cells and corresponding tumor tissue were characterized by mesothelial protein and gene expression analysis. In addition, primary cultures from chemo naive patients were exposed to increasing doses of cisplatin/pemetrexed during three months and compared with non-treated cells in a cytotoxicity assay, and by selected profiling of senescence markers. *In vitro* chemoresistance in the primary mesothelioma cell cultures was associated with increased Thy1 (CD90) expression. Thy1 expression in MPM samples was significantly associated with poor overall survival in the TCGA MPM cohort. Our results illustrate that the establishment of a large live-cell MPM biobank contributes to a better understanding of therapy resistance observed *in vivo*, which eventually may lead to a more logical approach for developing new treatment strategies.

## Introduction

Malignant pleural mesothelioma (MPM) is an asbestos-related cancer arising from mesothelial cells of the pleura. The prognosis of MPM patients is poor [median survival of 12 months (1)] likely because in most cases diagnosis is made in advanced disease stages. A recent large-scale study based on pathological samples has comprehensively characterized most genetic alterations and distinct molecular profiles in MPM (2). However, there is still the need of experimental models to define cancer driver activity of a given genetic alteration, like it has been done with the *Nf2*-(3–5) and *Bap1*-deficient mice (6, 7). Experimental models may closely resemble *in vivo* conditions and allow for the testing of novel therapeutic approaches to understand their mechanism of action.

Moreover, experimental models allow for the investigation of resistance mechanisms. Patient-derived xenografts in humanized NSG mice (https://www.jax.org/news-and-insights/jax-blog/2015/april/the-next-big-thing-in-cancer-modeling-patient-derived-xenografts-in-humaniz) would represent the best overall model but currently are difficult to implement for routine purposes. In contrast, cell lines are an easy accessible disease model (8) that is still accepted for certain investigations, such as molecular mechanism of drug action. Primary mesothelioma cultures were shown as valid model for mechanisms of resistance to pemetrexed, which, in combination with platinum compounds, is a component of current MPM standard neoadjuvant treatment (9). According to Cellosaurus (http://web.expasy.org/cgi-bin/cellosaurus/search) there are 402 human mesothelioma cell lines available. Of these cell models, only 11 have a well-profiled genotype (https://portals.broadinstitute.org/ccle/home) and for 45, fingerprinting for identification is available. In addition, long-term passaging leads to genetic drift caused by genomic instability. A recent study (10) demonstrated an upregulation of glycolytic and oxidative phosphorylation in commercial lines compared to primary cultures. In addition, primary cells maintain expression of podoplanin, which is lost in commercial lines and they show activation of the type I interferon signaling pathway (10).

In this study, we tested the hypothesis whether molecular profiling of primary mesothelioma cell culture obtained from patients with MPM are helpful to understand the mechanisms of acquired resistance to cisplatin/pemetrexed treatment *in vivo*.

## Materials and Methods

### Tumor

Tissue samples were obtained from patients treated for MPM at the Department of Medical Oncology and Department of Thoracic Surgery between January 2007 and December 2014. The study has been approved by the Zurich Cantonal Ethics Committee (reference numbers StV 24-2005 and 29-2009). Written informed consent was obtained from all patients. Tumor samples obtained from diagnostic biopsies prior to neoadjuvant chemotherapy and the corresponding resection specimens were processed immediately for total RNA extraction using Qiagen RNAeasy kit (Qiagen, Hilden, Germany). In addition, another part of the tumor samples was embedded in OCT and immediately frozen at −80°C. The remaining tumor samples were fixed in 4% buffered formaldehyde for paraffin embedding.

### Cell Culture and Senescence-Associated-β-Gal Activity

Primary mesothelioma cell cultures were generated as previously described (11). In order to establish a model for chemoresistance, two primary cultures from chemotherapy-naive epithelioid MPM specimens were continually exposed to low doses of cisplatin and pemetrexed according to a previously published method of cisplatin-resistance in ovarian cancer cell lines (12). These two cultures originated from two MPM patients. The first primary culture was taken from a biopsy of a male 69-year-old patient (P95), who had an epithelioid MPM (Figures S1 and S2 in Supplementary Material) and was enrolled into the SAKK17/04 study (13). The patient, therefore, underwent extrapleural pneumonectomy after neoadjuvant chemotherapy but relapsed 15 months later. He died 27 months after the initial diagnosis. The second culture was established from a female, 50-year-old patient (P236) with papillary epithelioid tumor morphology (Figure S1 in Supplementary Material). The patient underwent partial pleurectomy (Figure S2 in Supplementary Material) but relapsed 19 months later and was operated again, followed by chemotherapy. Unfortunately, the patient died 3 years after the first surgery. In the two chemo-naive primary cultures, cells were treated with cisplatin at the initial dose of 3 and 1 nM pemetrexed. The treatment was conducted in three cycles, including 4 days of treatment and 3 days of recovery time. The concentration was doubled and the procedure was repeated until the concentration of cisplatin and pemetrexed reached 48 and 16 nM, respectively. Chemoresistance induction was evaluated based on the cytotoxicity to a subsequent exposure to cisplatin and pemetrexed. Doubling time was estimated as previously described (14). Primary cells were authenticated by DNA fingerprinting of short tandem repeat loci (Microsynth, Switzerland). Senescence-associated-β-gal activity was determined as previously described (15).

### Relative Gene Expression

To extract RNA, Qiagen RNeasy kit was used. cDNA was determined as previously described (15). Selected gene expression [primers are listed in Table S1 in Supplementary Material and (15–17) analysis using MIQE compliant protocols (18)] was conducted as previously described.

### Western Blotting

Whole cellular protein extracts were prepared in 95°C Laemmli sample buffer and mechanically sheared by pressing few times through syringes (26 G). Protein concentration was determined using a Pierce™ 660 nm Protein Assay (Thermo Scientific). A total of 5 µg protein per extract was separated on denaturing 10–20% gradient SDS-PAGE gels. Proteins were transferred on PVDF transfer membranes (0.45 µm, Perkin Elmer). Membranes were probed with the following primary antibodies: anti-calretinin (Sigma, HPA007306), mouse anti-actin (#69100) from MP Biomedicals, N-Cadherin (BD Biosciences, 610920), YAP (Cell Signaling 4912), Mesothelin (Rockland Inc. 200-301-A88), GATA4 (C-20) (Santa Cruz sc-1237), p62 (Progen GP62-C), LC3B (Cell Signaling 2775S), p53 (DO-1) (Santa Cruz sc-126), Thy1 (H-110) (Santa Cruz sc-9163), and γ-H2AX (Millipore 05-636). Membranes were then incubated with the secondary antibody rabbit anti-mouse IgG-HRP (A-5420) from Antell, and goat anti-rabbit IgG-HRP (#7074) from Cell Signaling. The signals were detected by enhanced chemiluminescence (ECL™ Western Blotting Reagents, GE Healthcare) and detected on photosensitive film (Super RX Fuji x-Ray Film, Fujifilm). Proteins were quantitated with densitometry using Image J software (Version 1.42q, USA).

### Immunofluorescence

Primary cells were grown on 12-mm glass coverslips in 24-well plates. Cells were fixed for 10 min with 4% paraformaldehyde and permeabilized with 0.05% saponin for 5 min. The cells were then incubated over night at 4°C with Calretinin (N-term) (Sigma HPA007306), Podoplanin (D2-40) (Dako M3619), YAP1 (Cell Signaling 4912), Ki67 (Abcam ab8191), Phalloidin 488 (molecular probes A12379), Thy1 (H-110) (Santa Cruz sc-9163), and N-Cadherin from Dako (M3613) diluted in PBS containing 1% bovine serum albumin. Secondary antibodies, Alexa Fluor 488-conjugated goat anti-rabbit IgG (Life Technologies A11034) and Alexa Fluor 555-conjugated goat anti-mouse IgG (Life Technologies A21424) antibody was added for 1 h at RT. Nuclear DNA was stained using DAPI. Coverslips were mounted using Prolong Gold antifade reagent (Life Technologies). Images were acquired using an Olympus BX61 microscope (Schwerzenbach, Switzerland) equipped with an F-view camera for conventional fluorescence imaging. The image capture was controlled with the AnalySISPro software (Soft Imaging System, Münster, Germany).

### Surface Phenotyping

Cells resuspended in ice-cold PBS/2mM EDTA were incubated with antibodies against Thy1 (H-110) (Santa Cruz sc-9163) at 4°C for 30 min in the dark. Secondary antibody (PE-labeled, Dianova) was incubated at 4°C for 30 min in the dark. After washing with PBS cells were resuspended in ice-cold PBS/2mM EDTA and fluorescence was measured on an Attune flow cytometer (Applied Biosystems) and analyzed with the Attune cytometric software v1.2.5 (Applied Biosystems).

### Genetic Alterations

For DNA isolation from formalin-fixed, paraffin-embedded (FFPE) tissue, two punches of 0.6 mm were taken from the tumor of each FFPE Block. DNA was extracted using the Maxwell 16 FFPE Tissue LEV DNA Purification Kit (Promega) according to the manufacturer’s protocol. Isolated DNA was diluted in nuclease-free water and the concentration was measured by fluorometric quantitation using the Qubit 2.0 fluorometer (Thermo Fisher Scientific).

For targeted amplicon-based sequencing, we developed a custom-designed Ion AmpliSeq MPM Panel (Thermo Fisher Scientific), covering the coding regions of 30 genes that were reported to be commonly mutated in MPM. 10 ng of either FFPE tumor or primary cell DNA were used as an input for library preparation, following the Ion AmpliSeq DNA Library Preparation user guide (Thermo Fisher Scientific). Template preparation and sequencing was performed according to the manufacturer’s protocol (Ion PI Hi-Q OT2 200 Kit User Guide and Ion PI Hi-Q Sequencing 200 Kit, Thermo Fisher Scientific). Alignment, variant calling, and filtering of the resulting data were performed with Ion Reporter 5.0 (Thermo Fisher Scientific). The filtering chain included removal of all synonymous mutations, variants that are found in the UCSC Common SNPs database, variants with an allele ratio below 10% or a read count below 50. Additionally, all mutations were visually inspected, using the Integrative Genomics Viewer Software (Broad Institute).

For array-based genome wide copy number analysis, OncoScan FFPE microarrays (Affymetrix) were conducted at IMGM Laboratories GmbH (Martinsried, Germany). An input amount of 79.2 ng DNA was used for the assay. Molecular inversion probe processing was carried out according to the OncoScan FFPE Assay Kit Protocol (Affymetrix). For data analysis, the AGCC Viewer v4.2.1567 and the OncoScan Console v1.3.0.39 were used (Affymetrix). Copy number variation (CNV) and loss of heterozygosity (LOH) analysis was performed using the Nexus Express for OncoScan v3.1 software. CNVs were visualized using Circos19. Oncoscan have been deposited in GEO (https://www.ncbi.nlm.nih.gov/geo/query/acc.cgi?acc=GSE109305).

### Statistics

Data are expressed as mean ± SD. Statistical analysis was performed using Mann–Whitney *U* tests using StatView 5.0.1 (SAS institute) and *t*-test using GraphPad. Differences were considered statistically significant at *p* < 0.05.

## Results

### Primary Cultures Reflect Tumor Characteristics

**Figure 1:**
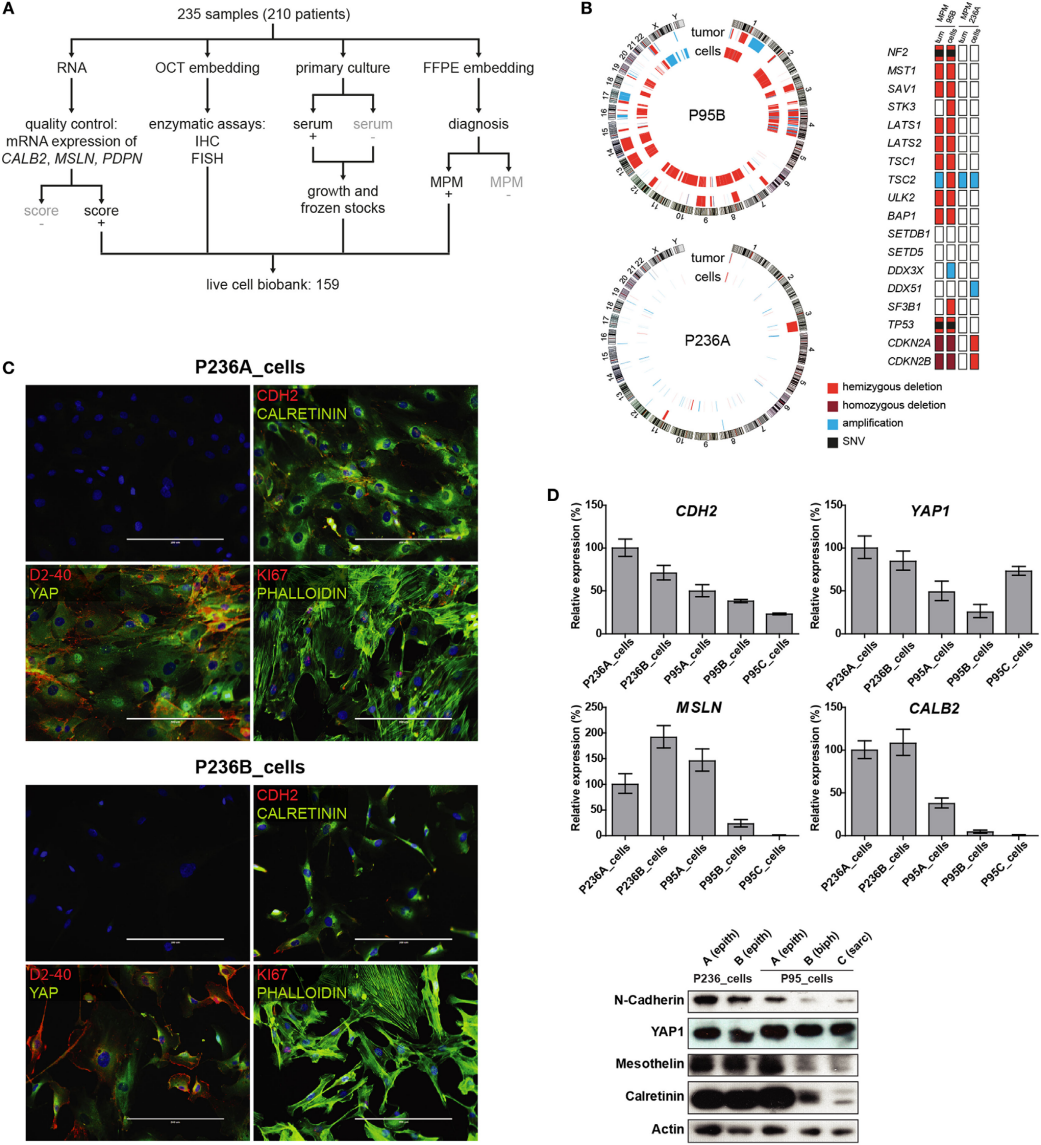

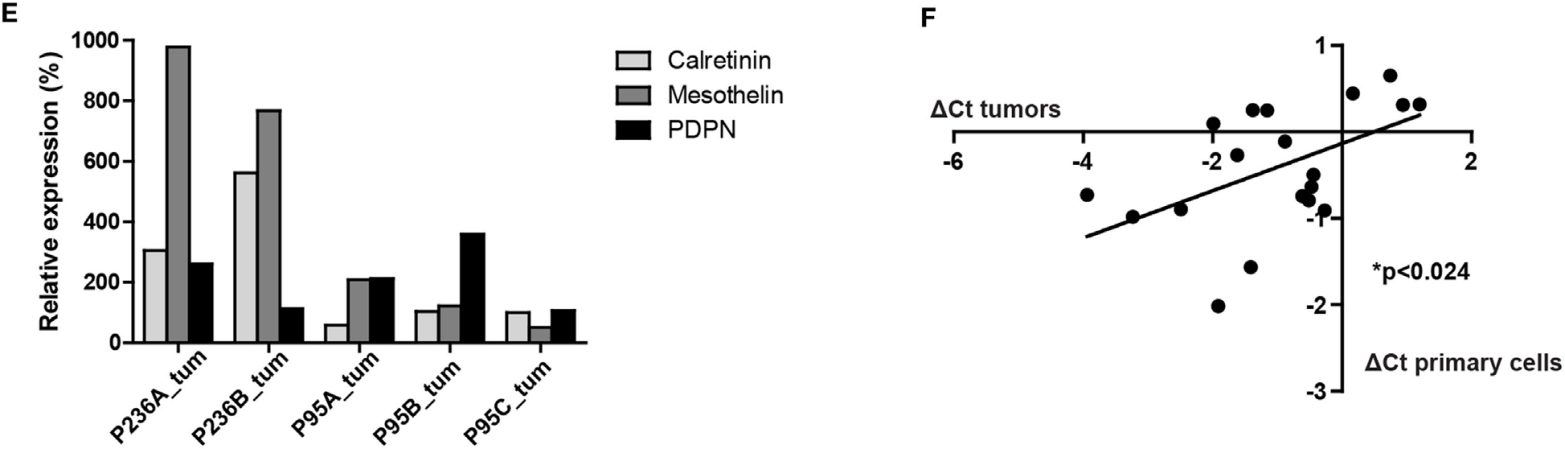
**(A)** Tumor processing flow-chart of 235 samples, which were processed for RNA extraction, cell culture and embedding either in PFA or OCT. Samples showing attributes which are grayed in the chart, were not used in the live cell biobank. **(B)** Circos whole genome copy number variations (CNVs) view of tumor and primary cell culture in patients malignant pleural mesothelioma (MPM) 236 and MPM95. The quilt plot highlights the CNV and SNVs in genes that are part of MPM landscape (2). **(C)** Immunofluorescence analysis of selected markers in primary culture from patient MPM236. Scale bar 200 µm. **(D)** Selected genes expression analysis at mRNA (upper panel) and protein (lower panel) level in primary cultures derived from samples from two patients, MPM236 and MPM95. The latter one underwent EMT during disease progression. **(E)** Selected genes expression analysis at mRNA in tumor samples from patients MPM236 and MPM95. **(F)** Significant correlation between gene expression changes in tumor and primary culture from patient MPM236 at passage 3.

Between 2007 and 2014, 235 surgical samples were obtained and processed, as described in Figure 1A. After preservation of tumor material for histopathological evaluation, remaining parts of the tumor were processed in order to extract RNA. Derived cDNA was then used for quality control assays, i.e., for calculation of the MPM score (15), allowing to determine that 82% of the samples contained at least 50% tumor cells. In case of large representative tumor tissue, additional parts of the fresh sample were used to establish primary cultures, either in the presence of serum (11) or in the absence of serum (17), for enzymatic analysis (15) or *in situ* hybridization (17). Successful primary cultures from “*bona fide*” tumor samples, defined as sufficiently growing cells for collecting RNA and total protein lysates, and preparing frozen stocks, was achieved in 68% of initial samples (159/235).

Primary cultures from two patients (P236 and P95) for whom samples were available at different stages of the disease (for nomenclature defining the different samples from these patients see Figure S2 in Supplementary Material) and where we had observed maintenance of epithelioid histology in one (P236) and epithelial to mesenchymal transition (EMT) in the second (P95), were selected for this proof of principle study. It is worth noting that EMT was evaluated on surgical specimens excluding any diagnostic bias introduced by single biopsy analysis. For these two patients, established primary cultures from different time points were compared with matching initial tumor samples by short tandem repeat analysis (Table S2 in Supplementary Material), Oncoscan CNV testing, and amplicon panel sequencing (Figure 1B). While few genetic alterations were present in P236, consistent with current knowledge on papillary mesothelioma (19), P95 was characterized by a widespread LOH including *NF2, BAP1*, and *TP53* tumor suppressor genes, as well an additional truncating mutation in *NF2* (c.553G > T/p.Glu185Ter, no Cosmic annotation) and a point mutation in *TP53* (c.839G > C/p.p.Arg280Thr, cosm254987).

N-cadherin, mesothelin, and calretinin MPM biomarker mRNA and protein expression was determined (Figure 1C) and decreasing calretinin expression reflected the evolution of the tumor. A high level of YAP expression was observed in all cultures. Culture heterogeneity was evidenced in one of the patient (P236_cells) by immunofluorescence analysis, revealing that not all cells stained for podoplanin (D2-40), Ki-67, nuclear YAP, or nuclear calretinin, while all cells expressed N-cadherin (Figure 1D). Changes in mesothelin, calretinin and podoplanin gene expression levels were also evaluated in tumor samples (Figure 1E). Differences observed *in vivo* for selected mRNAs involved either in differentiation (*HES1, RUNX2*), EMT (*GREM1, CTGF*), senescence (*PUMA, LOX, PAI1*), drug transport (*SLC22A4, ABCC1*), cell cycle (*CALB2* (20), *CCND1, CDKN2A*), response to oxidative stress or hypoxia (*HO-1, CAIX, GLUT1*), or MPM-associated surface proteins (*PDPN, THY1, CD276, TNFRSF10B*) were weakly (*R*^2^ = 0.2641) but significantly correlated with differences observed in primary cultures in patient P236 (Figure 1F). This supports the idea of using primary cultures for the functional investigation of mechanisms underlying tumor evolution.

### Development of Chemoresistance Is Accompanied by Increased Levels of DNA Damage, Autophagy and Senescence Marker β-Galactosidase

**Figure 2:**
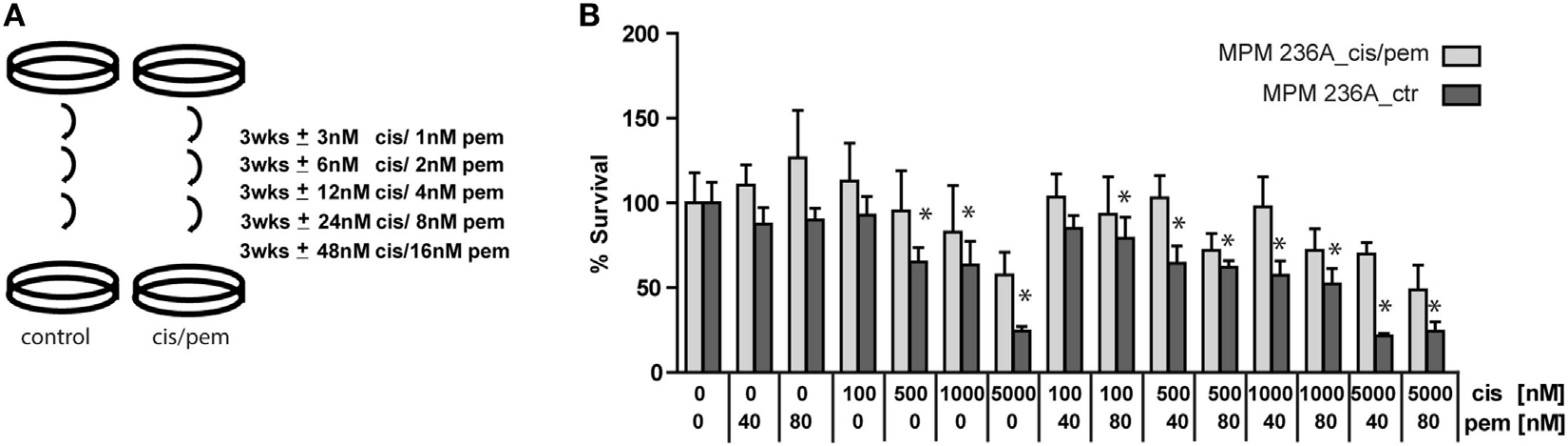
**(A)** Establishment *in vitro* of chemoresistant cell lines by exposure to gradually increasing doses of cisplatin/pemetrexed. **(B)** Cis/pem line established from patient P236A_cells is chemoresistant compared to control when challenged with pemetrexed/cisplatin. Two-way ANOVA with Bonferroni’s *post hoc* test, *p < 0.01.

Malignant pleural mesothelioma chemoresistance models have been described after 96 h exposure of human mesothelioma cell lines to high doses of pemetrexed (9) or after 15 cycles of combination of treatment with IC50 concentrations of cisplatin plus pemetrexed during 72 h followed by a recovery time in a rat cell line (21). We opted for a method that has been used in a study inducing cisplatin-resistance to ovarian cancer cell lines (12) (Figure 2A) to avoid acute induction of senescence-associated growth arrest observed using high doses of pemetrexed (9). Using this approach, we successfully generated a chemoresistant model for primary cells derived from patient P236 (Figure 2B) but not for primary cells derived from patient P95 (Figure S3 in Supplementary Material). This was accompanied by a higher basal chemoresistance in primary cells derived from patient P95 (IC50 2.5 vs. 1.5 µM in the primary culture from patient P236). Doubling time in the cultures exposed or not to cisplatin/pemetrexed did not differ in either of the two patients (Figure S4 in Supplementary Material) excluding that growth kinetics were the underlying mechanism of resistance.

**Figure 3:**
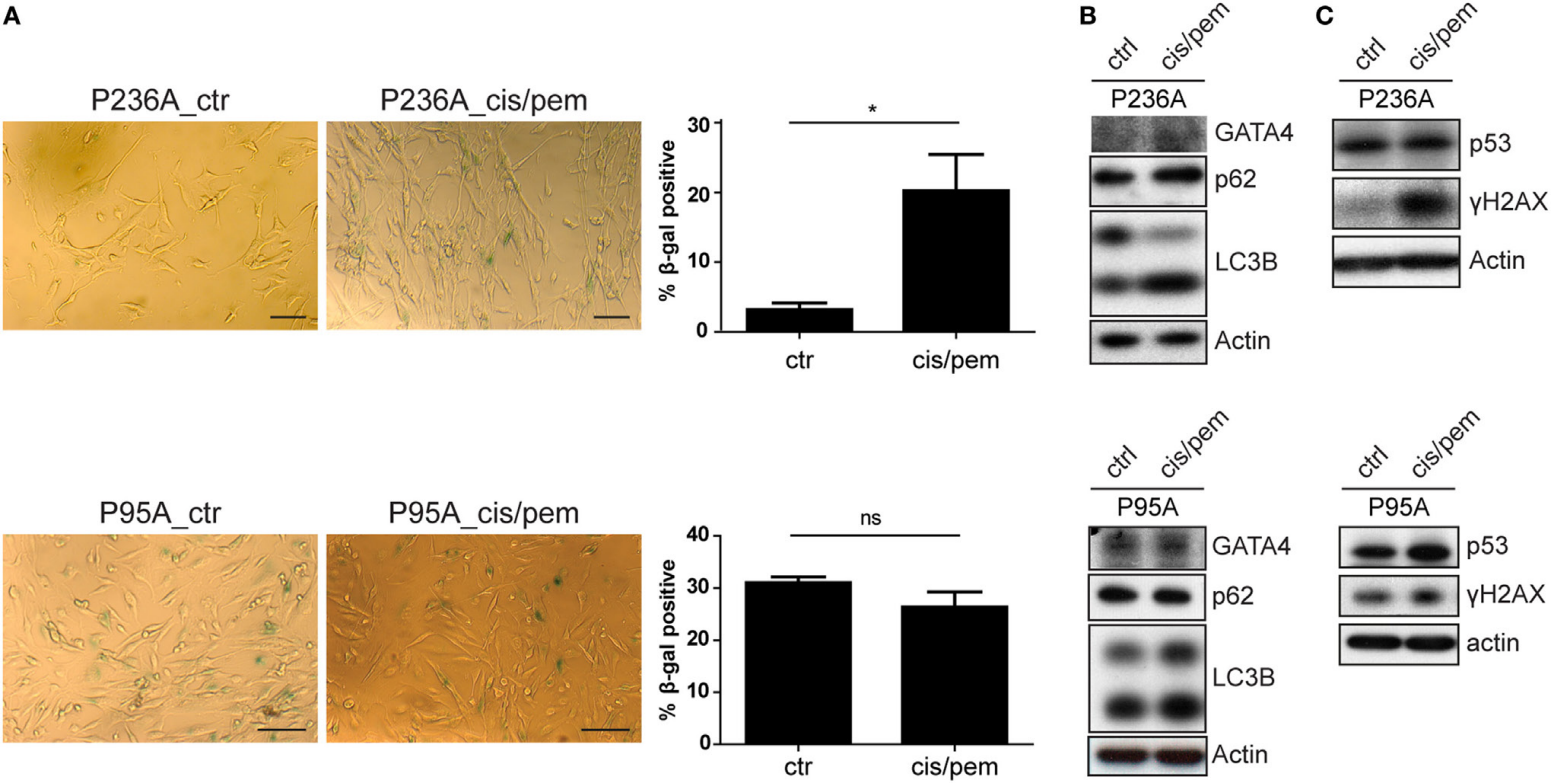
**(A)** Senescence-associated beta-galactosidase staining in control and cis/pem adapted primary cultures from patient malignant pleural mesothelioma (MPM) 236 and MPM95. Scale bar 25 µm. **(B)** Expression of senescence-associated secretion phenotype-associated GATA-4 and autophagy activation LC3B and p62. **(C)** DNA-damage response markers γ-H2AX and p53 stabilization.

We have previously described the occurrence of senescence in MPM patients treated with chemotherapy (15). Because senescence is a stress response involving multiple effector mechanisms, including DNA damage response (22, 23), senescence-associated secretion phenotype (SASP) (24–28) and autophagy (29), we analyzed markers involved in these pathway. Consistent with development of chemoresistance in the cisplatin/pemetrexed primary culture from patient P236, we observed increased levels of senescence-associated β-gal activity (Figure 3A), autophagy markers LC3B-II (53% increase) and p62 (41% increase) and appearance of SASP-associated (28) GATA-4 (Figure 3B), and H2AX phosphorylation (more than sevenfold increase, Figure 3C). Importantly, basal levels of senescence-associated β-gal activity were higher in patient P95, and did not differ in cisplatin/pemetrexed treated cultures. In the same patient the expression of GATA-4 was already detected in the control primary culture and did not increase further in the cisplatin/pemetrexed culture. Increased levels of the other markers in the cisplatin/pemetrexed compared to control culture were smaller compared to changes observed in patient P236. All in all, this data confirms that chemotherapy can induce resistance *via* induction of senescence but also indicates that the cancer genetic background *per se* is likely responsible for senescence and resistance due to genomic instability.

### Increase in Thy1 Expression in Acquired-Chemoresistance Line

**Figure 4:**
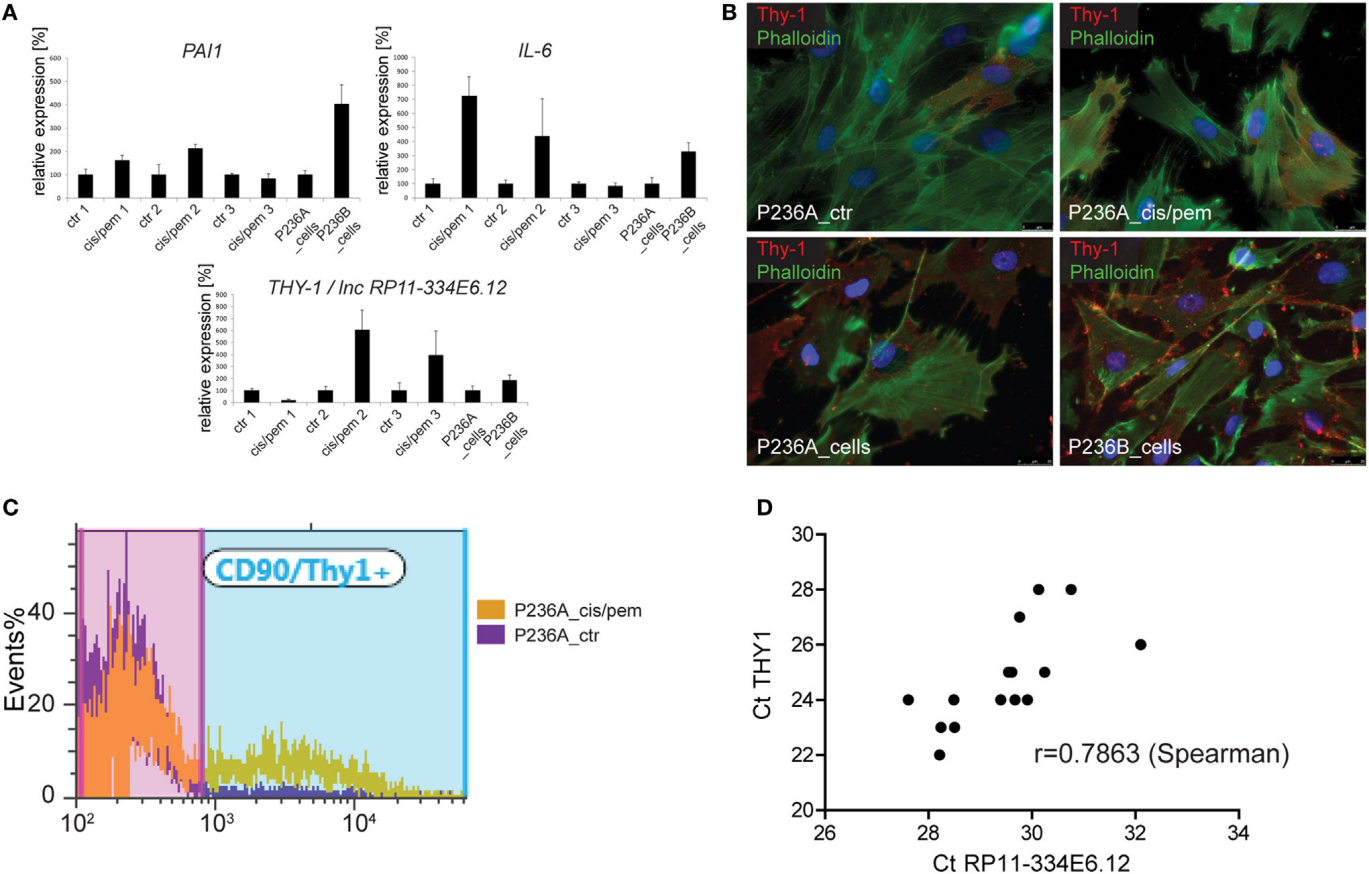
**(A)** Expression of senescence-associated genes *IL-6* and *PAI-*1 and *THY1/*lnc *RP11-334E6.12* during chemoresistance development or tumor progression. **(B)** Immunofluorescence analysis of Thy1 expression cis/pem and control lines and original primary cultures from tumor samples of malignant pleural mesothelioma (MPM) 236 during tumor progression. **(C)** Flow cytometry analysis of Thy1 expression in cis/pem vs. control line. **(D)** lnc *RP11-334E6.12* mRNA expression correlates with *THY1* expression in MPM tumors.

In order to get more insight into events occurring during the development of chemoresistance in primary cultures from patient P236 exposed to cisplatin/pemetrexed, we analyzed the mRNA expression of autophagy/senescence-associated *IL-6* and *PAI-1* (15, 30) and chemoresistance-development associated Thy1 (31) at three different time points during culture treatment (Figure 4A) and in the original primary cultures. Consistent with increased level of SASP-associated GATA4, we observed upregulation of *IL-6* at the first two time points and in the primary culture obtained in the progressing tumor. *PAI-1* expression followed a similar pattern. *THY1* was decreased at the first time point then it increased, as also observed in the progressing tumor. We had identified Thy1 N-glycoprotein on the cell surface of mesothelioma cells in MPM tumors (32), therefore, we analyzed Thy1 positive cells by immunofluorescence in both, control and cisplatin/pemetrexed adapted primary cultures and original primary cultures from the two specimens obtained from the tumor. We observed that Thy1 was expressed at increased levels in primary cultures obtained during cisplatin/pemetrexed adaptation (Figure 4B, upper panel) and as well during tumor progression (Figure 4B, lower panel), although to a lower extent. For quantification, we performed flow cytometric analyses (Figure 4C) and observed a 10-fold increase of expression of Thy1 surface protein in cisplatin/pemetrexed exposed primary cells at the third time point, confirming our immunofluorescence data. In addition, we detected Thy1 protein expression by WB (data not shown) in cisplatin/pemetrexed adapted cells. The observation associating higher levels of Thy1 with tumoral cell progression is consistent with Thy1 high expression levels with worst overall survival in TCGA (Figure S5 in Supplementary Material).

In the process of trying to better understand mechanisms regulating Thy1 expression, we realized that we had used primers (15) based on UCSC Genome Browser on Human Feb. 2009 Assembly, which detect both *THY1* and long non-coding RNA (lncRNA) *RP11-334E6.12*, a lncRNA in antisense orientation compared to *THY1* and which partially overlaps with *THY1* 3′UTR. We, therefore, redesigned primers recognizing individually *THY1* and lncRNA *RP11-334E6.12*, and observed a strong correlation between lncRNA *RP11-334E6.12* and *THY1* expression (Figure 4D), which is consistent with correlated expression between these two gene expression reported in G.Tex (33) (Figure S6 in Supplementary Material). Taken together, this data indicate an enrichment of THY1 positive cells during chemoresistance development and a possible role for lnc *RP11-334E6.12* in such an increase.

## Discussion

A national effort in UK has resulted in the establishment of 26 primary mesothelioma cultures within the UK Mesobank (34). In this study, we tested the usefulness of an even larger collection of primary mesothelioma cell cultures, in some cases obtained from the same patient at different stages during disease progression. We aimed to carry out functional studies and provide evidence that changes observed *in vitro* and *in vivo* are similar.

Primary cultures in cancer research are of high value. Indeed, they represent primary intermediate before growth as xenograft, which, although providing a three-dimensional tissue environment have high-cost and low-throughput capacity. We have highlighted (17) the importance of primary culture and their growing conditions for maintenance of stem cell signaling pathways active *in vivo* (2, 35). In addition, they may represent the unique resource available for the 60% of MPM that do not grow as xenograft (36).

Although high-quality repositories such as biobanks and biomolecular resource infrastructures are being established (37), access to a large numbers of MPM biospecimens of high quality remains difficult. In our study, we deeply characterized samples from two patients, exemplifying the heterogeneity that can be found in real life, with one patient showing high levels of chromosomal instability while in the second only minor alterations were observed.

In the primary culture from the patient with abundant chromosomal instability (P95), a basal level of chemoresistance associated with senescence-associated phenotype was observed. This is consistent with the presence of the gain of function p53 mutation observed in this patient. Indeed p53Arg280Threo, although not fully oncogenic, has been shown to increase growth in soft agar of p53-deficient cells (38). *TP53* is rarely mutated in MPM and in a recent high-through put study (2) mutations in this gene were absent in epithelioid MPM. However, datamining the catalog of somatic mutations in cancer (COSMIC, http://www.sanger.ac.uk/cosmic), we found that *TP53* mutations are described in 2/24 (8%) epithelioid mesothelioma. For this patient, we have already described that the side population was enriched in tumor initiating properties in the primary culture established at the time of surgery, where tumor has evolved from epithelial to biphasic histotype (35), and Hedgehog signaling is maintained in primary cultures in serum-free conditions (17). Because we now show that a resistant phenotype against cisplatin/pemetrexed is present before chemotherapy, we infer that intrinsic properties of the tumor, including gain of function of *TP53*, deletion of *NF2*, and *CDKN2A* participate to the evolution of resistant cells toward EMT.

The tumor from the second patient (P236) was of epithelioid morphology, maintaining the same histotype throughout the course of the disease and displayed absence of gross genetic abnormalities. In this context, it is worth noting that in triple negative breast cancer, 26% of cancers lack gross genetic abnormalities and this is likely because they originate from an epithelial precursor with stemness properties (39). The second patient was operated again after relapsing and received chemotherapy after the second surgery but relapsed again.

Primary mesothelioma cultures obtained at a 4-year interval have already been shown to demonstrate increased drug resistance but underlying mechanisms have not been examined yet (40). While investigating mechanisms underlying the development of chemoresistance, we observed increased Thy1 (CD90) expression. Thy1 is GPI-anchored cell surface protein and in human the highest expression is observed in fetal thymic cells and subgroup of fibroblasts (41). Thy1 overexpression suppresses cell proliferation in ovarian and nasopharyngeal cancer (42–46), however, Thy1 expression in cancer stem-cells has mostly been associated with chemoresistance and increased tumor-initiating properties (47–49). Thy1 expression has been also observed in four tumorigenic MPM primary cell cultures grown in low serum conditions (50). We show here that increased Thy1 was associated with increased expression of lncRNA *RP11-334E6.12*. There is not much information about this lncRNA. A significant correlation between expression of lncRNA *RP11-334E6.12* and developmental processes has been documented in esophageal cancer (51); overexpression has been documented in triple negative breast cancer (52). Because this lncRNA is in antisense orientation compared to *THY1*, and partially overlaps with *THY1* 3′UTR, it is tempting to speculate that it may participate in regulating THY1 expression. Although beyond the scope of this study, this hypothesis requires further investigation. In future studies, it would be of interest to investigate whether Thy1 expression contributes to chemoresistance and whether all MPMs are derived from a yet to be identified precursor cell.

## Ethics Statement

Zurich Cantonal Ethics Committee (reference numbers StV 24-2005 and 29-2009).

## Author Contributions

EF-B and PW designed experiments. KO, JK-R, and EF-B carried out experiments and interpreted data. KO, JK-R, and EF-B generated figures. KO and EF-B wrote the manuscript. All authors read and approved the final manuscript.

## Conflict of Interest Statement

The authors declare that the research was conducted in the absence of any commercial or financial relationships that could be construed as a potential conflict of interest.
