# Supplementary material for: Live-Cell Mesothelioma Biobank to Explore Mechanisms of Tumor Progression
Source: Front Oncol. 2018 Feb 23;8:40. doi: 10.3389/fonc.2018.00040 (PMC5829086; doi:10.3389/fonc.2018.00040)
Supplement: Supplementary file 1 [file Data_Sheet_1.DOCX]

Supplementary Material

Live-cell mesothelioma biobank to explore mechanisms of tumor progression

**Kathrin Oehl^1^, Jelena Kresoja-Rakic^2^, Isabelle Opitz^2^, Bart Vrugt^1^, Walter Weder^2^, Rolf Stahel^3^, Peter Wild^1^, Emanuela Felley-Bosco^2^***

*** Correspondence:** Emanuela Felley-Bosco

Laboratory of Molecular Oncology, Division of Thoracic Surgery, University Hospital Zürich, Sternwartstrasse 14, Zurich, Switzerland, P: +41 44 255 27 71
emanuela.felley-bosco@usz.ch

# Supplementary Figures and Tables

## Supplementary Figures


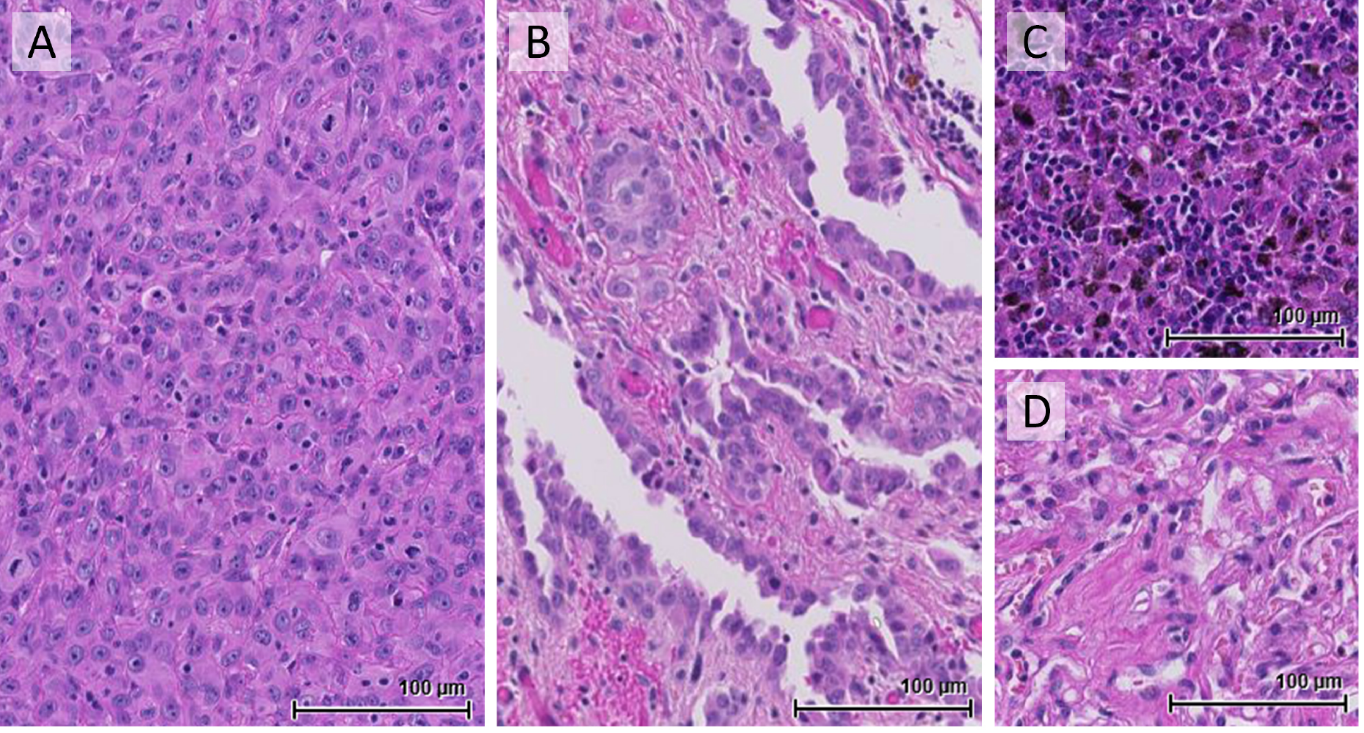


**Supplementary Figure 1.** Histology of chemo naïve tumor tissue (A: P95A_tum, B:P236A_tum) and the normal tissue controls (C: P95A_NT, D: P236A_NT).


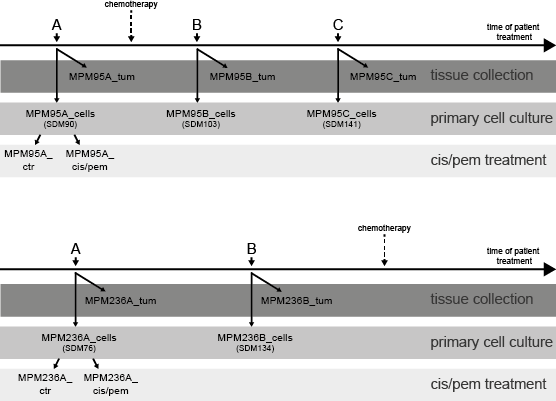


**Supplementary Figure 2**. Identity codes to define either tumors (tum) collected from a given patient (P) at different times (A, B, C), or primary cultures (cells), that were used for development of chemoresistance model (cis/pem) or maintained as control (ctr).


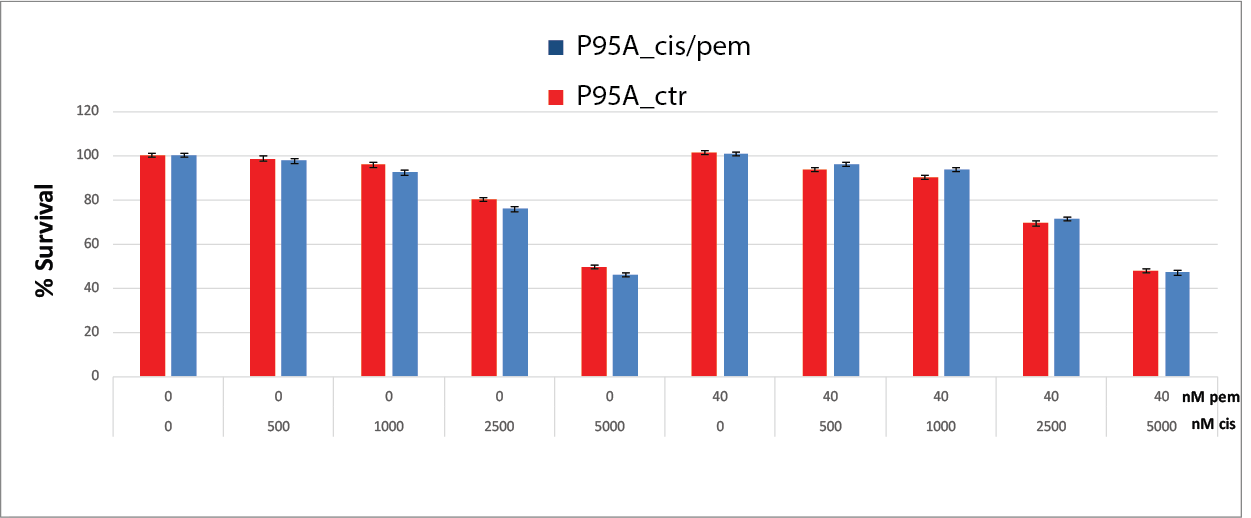


**Supplementary Figure 3**. Exposure of patient P95A_cells to gradually increasing doses of cisplatin/pemetrexed did not result in establishment of chemoresistance.


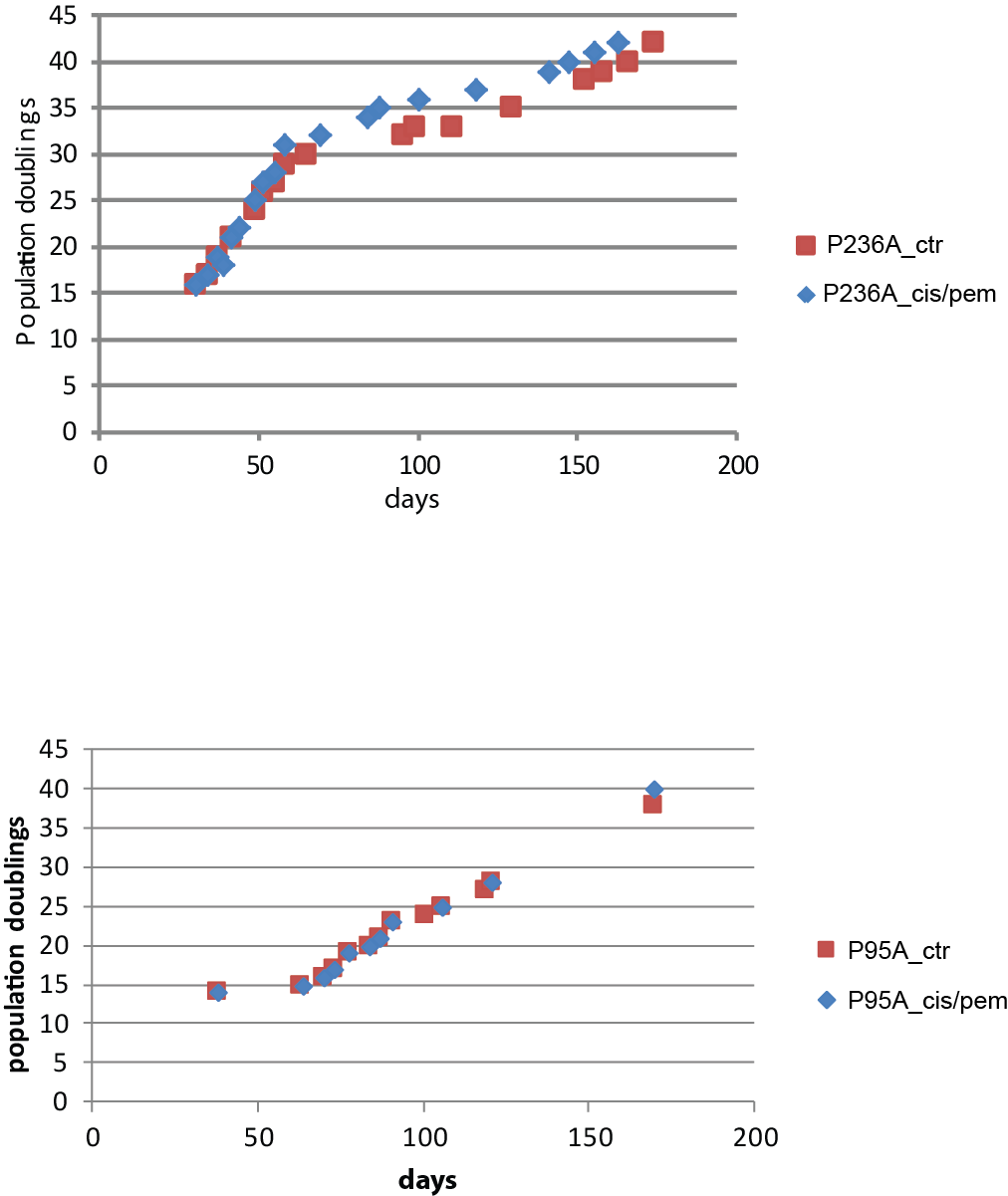


**Supplementary Figure 4**. No difference in population doublings was observed in cells exposed to gradually increasing doses of cisplatin/pemetrexed compared to the control line in P95A_cells or P236A_cells.


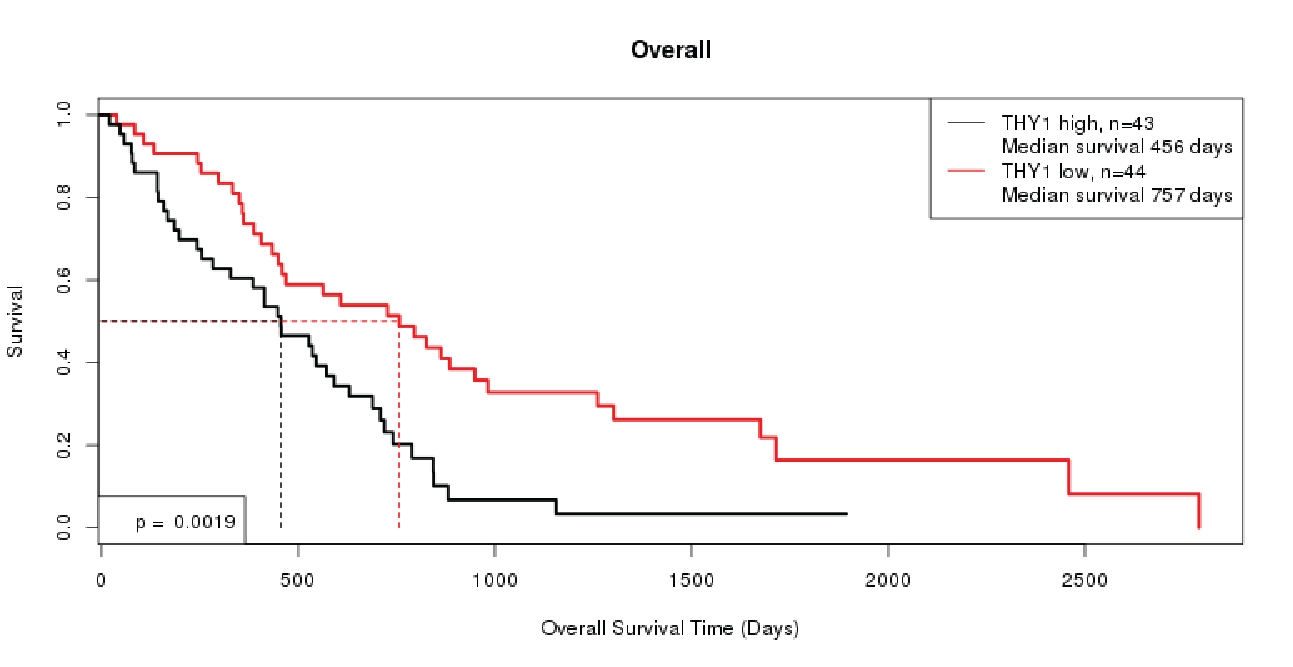


**Supplementary Figure 5**. *Thy-1* high expression levels are associated with worst overall survival in TCGA mesothelioma database.


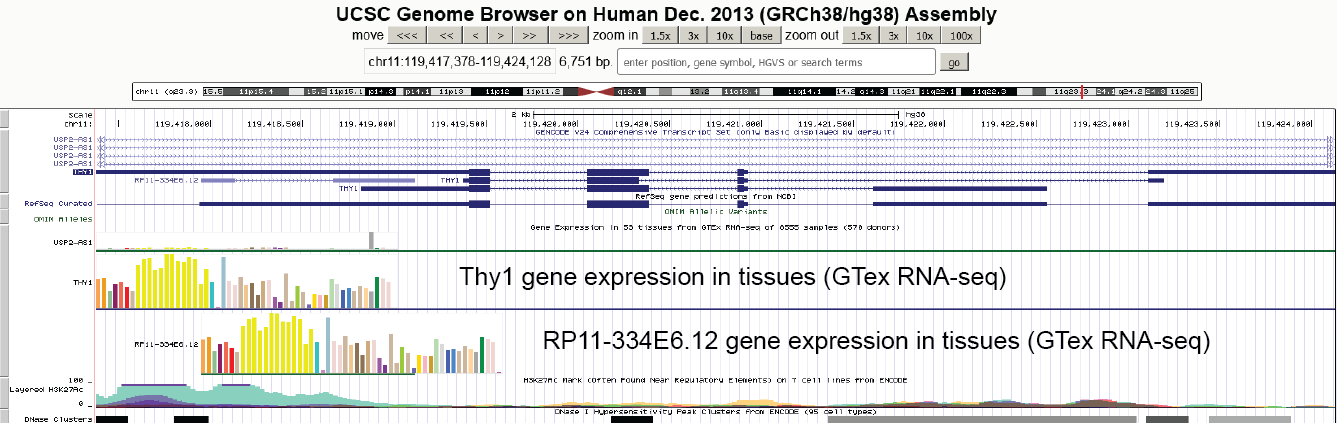


**Supplementary Figure 6**. *Thy-1* and *lncRNA RP11-334E6.12* expression levels are highly correlated in GTex database (UCSC Genome browser).

## Supplementary Tables

**Supplementary Table 1** List of primers used in the quantitative PCR analysis

| Gene name | GenBank  Accession  Number | Primer sequences |
| --- | --- | --- |
| IL-6 | NM_000600.4 | F: GCACTGGCAGAAAACAACCT  R: CAGGGGTGGTTATTGCATCT |
| Grem | AF11013 | F: CTTTCAGTCCTGCTCCTTCTG  R: CGTGTGACTCTCTTCTTCTTGG |
| CTGF | NM_001901.2 | F: TTGCGAAGCTGACCTGGAAGAGAA  R: AGCTCGGTATGTCTTCATGCTGGT |
| SLC22A4 | NM_003059 | F: TTTGGGATCACCTCTGCTTTCT  R: TGTTGTAAGCACCGAGGTAAACA |
| ABCC1 | NM_019898.2 | F: Gggctgcggaaagtcgt  R: agcccttgatagccacgtg |
| CD276 | NM_001024736.1 | F: AGAGCTGTGAGGAGGAGAAT  R: TGCTGTCAGAGTGTTTCAGAG |
| FOXO3 | NM_001455.3 | F: ACTCACTTAGCCACAGCGATGTCA  R: ATCATTGCGAAGCATCACGTTCCG |
| TXN | NM_003329.3 | F: AGAGAGCAAGCAGCGAGTCTTGAA  R: TCGATCTGCTTCACCATCTTGGCT |
| Pig3 | NM_004881.4 | F: ACCAAAGGTGCTGGAGTTAAT  R: CTCCTCCCATCAGACCATAGA |
| RP11-334E6.12 | ENST00000578216.1 | F: CAGCGAAGCGGGGGCAGGG  R: Ggccttggggcatgacccag |
| Thy1 (CD90) | NM_006288.4 | F: TGACCCGTGAGACAAAGAAG  R: GCTAGTGAAGGCGGATAAGTAG |

F, Forward primer; R, Reverse primer

**Supplementary Table 2.** STR profile of primary cultures derived from patient P236.

**P236A_cells**

| Locus | chromosome | | Typed allele |  |
| --- | --- | --- | --- | --- |
| D3S1358 | Chr03 | | 14/16 |  |
| TH01 | Chr11 | 6 | | |
| D21S11 | Chr21 | | 27/32.2 |  |
| D18S51 | Chr18 | | 13/14 |  |
| Penta_E | Chr15 | | 7 |  |
| D5S818 | Chr05 | 11 | | |
| D13S317 | Chr13 | 11/12 | | |
| D7S820 | Chr07 | 9/12 | | |
| D16S539 | Chr16 | 11/12 | | |
| CSF1PO | Chr05 | 11 | | |
| Penta_D | Chr21 | | 9/10 |  |
| AMEL | X/Y | X | | |
| vWA | Chr12 | 18/19 | | |
| D8S1179 | Chr08 | | 8/13 |  |
| TPOX | Chr2 | 10/11 | | |
| FGA | Chr04 | | 23/24 |  |

**P236B_cells**

| Locus | | chromosome | | Typed allele | |
| --- | --- | --- | --- | --- | --- |
| D3S1358 | | Chr03 | | 14/16 | |
| TH01 | | Chr11 | | 6 | |
| D21S11 | | Chr21 | | 27/32.2 | |
| D18S51 | | Chr18 | | 13/14 | |
| Penta_E | | Chr15 | | 7 | |
| D5S818 | | Chr05 | | 11 | |
| D13S317 | | Chr13 | | 11/12 | |
| D7S820 | | Chr07 | | 9/12 | |
| D16S539 | | Chr16 | | 11/12 | |
| CSF1PO | | Chr05 | | 11 | |
| Penta_D | | Chr21 | | 9/10 | |
| AMEL | | X/Y | | X | |
| vWA | | Chr12 | | 18/19 | |
| D8S1179 | | Chr08 | | 8/13 | |
| TPOX | | Chr2 | | 10/11 | |
| FGA | | Chr04 | | 23/24 | |
|  | |  | |  | |
|  |  | |  | |  |
|  | |  | |  | |

**P236A_ctr**

| Locus | chromosome | Typed allele |
| --- | --- | --- |
| D3S1358 | Chr03 | 14/16 |
| TH01 | Chr11 | 6 |
| D21S11 | Chr21 | 27/32.2 |
| D18S51 | Chr18 | 13/14 |
| Penta_E | Chr15 | 7 |
| D5S818 | Chr05 | 11 |
| D13S317 | Chr13 | 11/12 |
| D7S820 | Chr07 | 9/12 |
| D16S539 | Chr16 | 11/12 |
| CSF1PO | Chr05 | 11 |
| Penta_D | Chr21 | 9/10 |
| AMEL | X/Y | X |
| vWA | Chr12 | 18/19 |
| D8S1179 | Chr08 | 8/13 |
| TPOX | Chr2 | 10/11 |
| FGA | Chr04 | 23/24 |

**P236A_cis/pem**

| Locus | chromosome | Typed allele |
| --- | --- | --- |
| D3S1358 | Chr03 | 14/16 |
| TH01 | Chr11 | 6 |
| D21S11 | Chr21 | 27/32.2 |
| D18S51 | Chr18 | 13/14 |
| Penta_E | Chr15 | 7 |
| D5S818 | Chr05 | 11 |
| D13S317 | Chr13 | 11/12 |
| D7S820 | Chr07 | 9/12 |
| D16S539 | Chr16 | 11/12 |
| CSF1PO | Chr05 | 11 |
| Penta_D | Chr21 | 9/10 |
| AMEL | X/Y | X |
| vWA | Chr12 | 18/19 |
| D8S1179 | Chr08 | 8/13 |
| TPOX | Chr2 | 10/11 |
| FGA | Chr04 | 23/24 |
